# The role of m6A-associated membraneless organelles in the RNA metabolism processes and human diseases

**DOI:** 10.7150/thno.99019

**Published:** 2024-08-06

**Authors:** Fang-tian Bu, Hai-yan Wang, Chao Xu, Kang-li Song, Zhen Dai, Lin-ting Wang, Jie Ying, Jianxiang Chen

**Affiliations:** 1 College of Pharmacy and Department of Hepatology, Institute of Hepatology and Metabolic Diseases, the Affiliated Hospital of Hangzhou Normal University, Hangzhou Normal University, Hangzhou, Zhejiang 311121, China.; 2 Key Laboratory of Elemene Class Anti-Cancer Chinese Medicines; Engineering Laboratory of Development and Application of Traditional Chinese Medicines; Collaborative Innovation Center of Traditional Chinese Medicines of Zhejiang Province, Hangzhou Normal University, Hangzhou, Zhejiang 311121, China.; 3 Department of Gastroenterology, Affiliated Nanjing Jiangbei Hospital of Xinglin College, Nantong University, Nanjing 210044, P. R. China.; 4 Laboratory of Cancer Genomics, Division of Cellular and Molecular Research, National Cancer Centre Singapore, 169610, Singapore.

**Keywords:** *N*6-methyladenosine, membraneless organelle, RNA metabolism, liquid-liquid phase separation, human diseases

## Abstract

*N*6-methyladenosine (m6A) is the most abundant post-transcriptional dynamic RNA modification process in eukaryotes, extensively implicated in cellular growth, embryonic development and immune homeostasis. One of the most profound biological functions of m6A is to regulate RNA metabolism, thereby determining the fate of RNA. Notably, the regulation of m6A-mediated organized RNA metabolism critically relies on the assembly of membraneless organelles (MLOs) in both the nucleus and cytoplasm, such as nuclear speckles, stress granules and processing bodies. In addition, m6A-associated MLOs exert a pivotal role in governing diverse RNA metabolic processes encompassing transcription, splicing, transport, decay and translation. However, emerging evidence suggests that dysregulated m6A levels contribute to the formation of pathological condensates in a range of human diseases, including tumorigenesis, reproductive diseases, neurological diseases and respiratory diseases. To date, the molecular mechanism by which m6A regulates the aggregation of biomolecular condensates associated with RNA metabolism is unclear. In this review, we comprehensively summarize the updated biochemical processes of m6A-associated MLOs, particularly focusing on their impact on RNA metabolism and their pivotal role in disease development and related biological mechanisms. Furthermore, we propose that m6A-associated MLOs could serve as predictive markers for disease progression and potential drug targets in the future.

## Introduction

To date, more than one hundred chemical decorations on RNA have been discovered in eukaryotes 1. *N*6-methyladenosine (m6A), a methyl modification on adenosine, represents the most prevalent and dynamic post-transcriptional modification in eukaryotic mRNA as well as certain noncoding RNAs (ncRNAs) 2, 3. Commonly, m6A is installed by methyltransferases (writers), such as METTL3 and METTL16 4, 5, and removed by demethylases (erasers), such as ALKBH3, ALKBH5 and FTO 6, 7. Besides, readers preferentially bind to m6A determines the fate of mRNA. Different discovered readers, such as YTH (YT521-B homology) domain proteins 8, IGF2BP proteins 9 and HnRNP family proteins 10, are involved in RNA transcription, splicing, export, degradation and translation. Physiologically, the dynamic m6A modification plays a crucial role in germ cell meiosis 11, embryonic development 12 and immune response 13. However, destruction of the physiological levels of m6A leads to the emergence and development of various diseases, including infertility 14, neurodegenerative disease 15 and tumorigenesis 16. These unexpected effects are likely attributed to metabolic disorders of key mRNAs associated with m6A modification. Intriguingly, a substantial pool of mRNAs marked by m6A facilitates the formation of membraneless organelles (MLOs) or biomolecular condensates (BioMCs) formation through interacting with m6A writers, readers or erasers, which represents a crucial process in mRNA metabolism and turnover 16-18. Liquid-liquid phase separation (LLPS) promotes the assembly and formation of these m6A-assocaited MLOs or -BioMCs 18, 19. Many reports indicate that multivalent low-affinity interactions, encompassing RNA-RNA, RNA-protein and protein-protein, can trigger LLPS 20. Importantly, post-transcriptional modification, particularly m6A, provides “available bacon” for different RNA binding proteins containing internally disordered regions (IDRs) during the process of LLPS.

Recently, MLOs or BioMCs driven by m6A-modified RNAs have been proved to participate in transcription 21, alternative splicing (AS) 22, miRNA biogenesis 23, export 24, mRNA decay 18 and translation 25. These m6A-associated MLOs or BioMCs ensure efficient RNA metabolism under physiological conditions, thereby facilitating robust cellular homeostasis. Nevertheless, maladjustment of m6A modification, such as deficiency of ALKBH5, a crucial m6A demethylase, results in diminished nuclear speckle activity and dysregulated mRNA export and splicing in mammalian cells. This distinctively leads to impaired fertility in male mice although diverse biological functions of ALKBH5 have been revealed successively 6. Differently, MLOs driven by m6A modifications, such as nuclear YTHDC1-m6A condensates (nYACs), protect oncogenic mRNAs from degradation by polyA tail exosome targeting complex (PAXT), which contributes to the survival of cancer cells in acute myeloid leukemia 26. Moreover, excessive MLOs formation such as stress granules (SGs) is associated with tauopathy, and hnRNPA2B1 (a reader for m6A) mutation leads to Tau aggregation and sequesters itself in SGs 27. Although it has been established that m6A-associated MLOs play a regulatory role in various pathological processes, a comprehensive understanding of how these MLOs precisely affect specific biochemical processes, and the precise functional association between MLOs and the emergence and processes of different human diseases remains elusive.

In this review, we will outline the updated biochemical processes of m6A-associated MLOs, especially for RNA metabolism, and explore their implications in disease development and related biological mechanisms. Importantly, we put forward the hypothesis that the number of m6A-associated MLOs may predict the disease progression, and intervening in MLOs production or changing m6A levels will be an effective strategy to alleviate the disease process, especially for cancer cell growth.

## The selected biological reaction, substrate and significance of m6A modification

**Figure 1:**
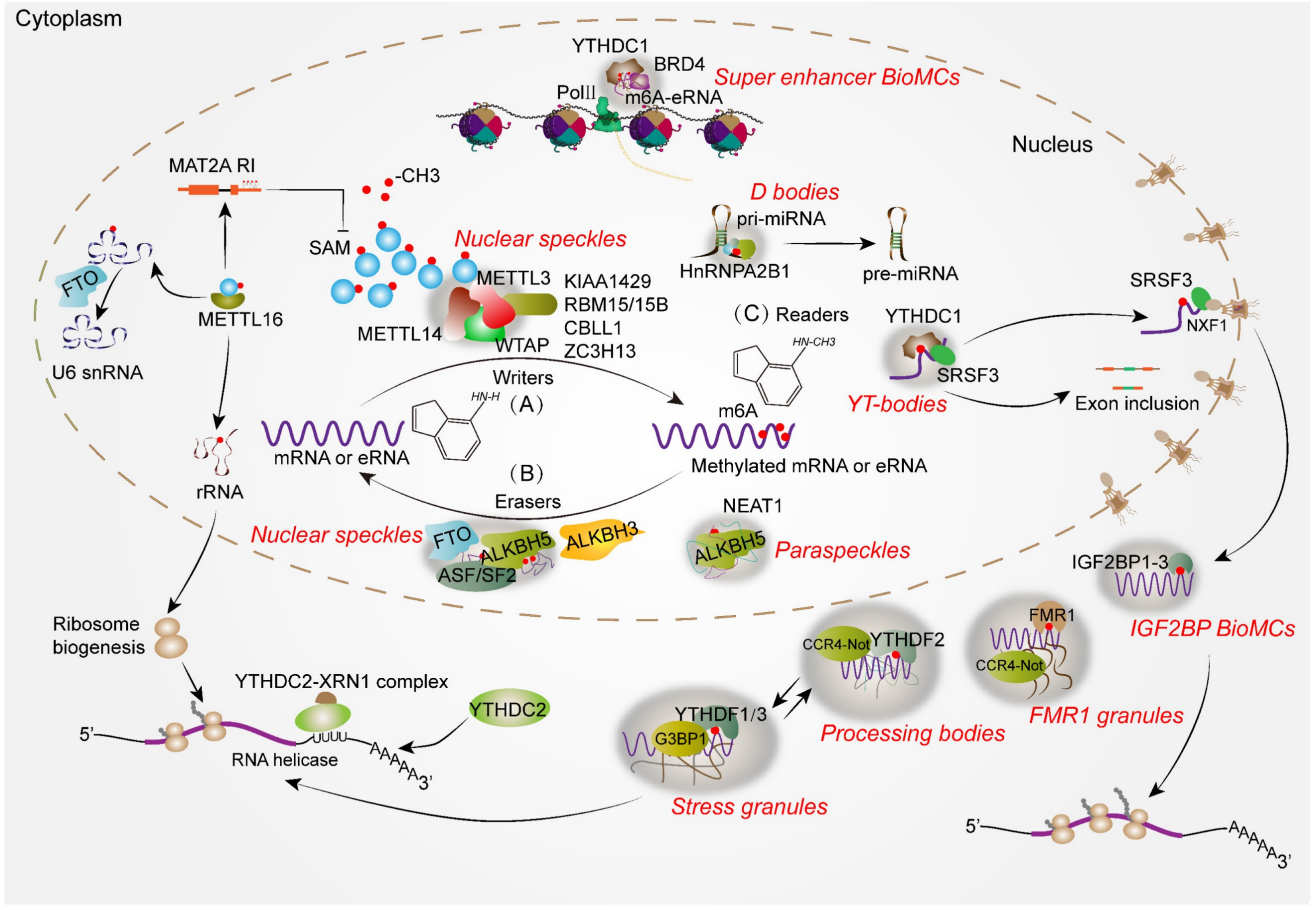
** m6A-associated MLOs or BioMCs run through the life of RNA. (A)** METTL16 controls cellular SAM levels through regulating the intron retention of SAM synthetase, MAT2A. Generally, m6A modifications on mRNA, eRNA or ncRNA are catalyzed by distinct methyltransferases such as METTL16 or “writer complex-MTC” comprising the METTL3-METTL14 heterodimer and essential adaptor proteins including WTAP, KIAA1429, RBM15/15B, CBLL1 and ZC3H13. In this process, METTL3 enhances the assembly of MTC through LLPS, while WTAP recruits METTL3-METTL14 heterodimer to NSs. METTL16 can promote the biogenesis of ribosome through methylating rRNA in the nucleolus, indirectly enhancing mRNA translation.** (B)** The removal of methyl groups is completed by demethylases (erasers), including FTO, ALKBH5 and ALKBH3. FTO and ALKBH5 are predominantly localized in NSs, where their demethylation activity facilitates RNA nuclear retention or alternative splicing. Besides, ALKBH5 promotes the assembly of PSs through LLPS and demethylating NEAT1, a vital scaffolding lncRNA for PSs formation, thereby enhancing its stability. **(C)** The fate of m6A-modified RNA largely depends on the formation MLOs or BioMCs mediated by different m6A readers, which serves as an indicator for RNA metabolism direction. YTHDC1 promotes the formation of nuclear MLOs to regulate RNA transcription, splicing and export. YTHDC1 forms YT-Bodies at transcription active sites and adjacent to NSs, defining splicing factor SRSF3 location in the NSs as well as enhancing its RNA-binding affinity, thereby promoting exon inclusion. In addition, the interaction between YTHDC1 and SRSF3 contributes to RNA crowding to NXF1, which is required for nuclear methylated mRNA export. Additionally, YTHDC1 drives the formation of super enhancer BioMCs through interacting m6A-modified eRNAs and BRD4, leading to enhanced RNA transcription. HnRNPA2B1 represents another type of m6A reader that regulates miRNA processing potentially by enhancing D-bodies formation through interactions with DGCR8 and DROSHA. Cytoplasmic m6A readers including YTHDF1-3, IGF2BP1-3 and YTHDC2 are implicated in regulating RNA stabilization, degradation and translation processes. YTHDF1-3 enhances the formation of SGs upon external stimuli. After stimuli removal, certain mRNAs combined with initiation factors will transport into the ribosomes for translation. Compared with YTHDF1/3, YTHDF2 colocalizes with both SGs and PBs, primarily mediating RNA degradation via interacting with CCR4-Not complex. FMR1 promotes maternal RNA degradation by forming FMR1 granules with m6A-modified RNAs and CCR4-Not. IGF2BPs are a group of m6A readers that enhance the stability of m6A-modified mRNA and further promote their translation through forming IGF2BP BioMCs.

Studies have found that a variety of chemical modifications, such as methylation, acetylation and phosphorylation, occur in DNA, RNA and protein, among which RNA is the most abundant substrate for chemical modification. More than 170 RNA chemical modifications have been identified yet, which are widely distributed in mRNA, tRNA, rRNA, spliceosomal RNA and other ncRNAs 1, 28. RNA methylation accounted for more than 60% of RNA modification, and the common RNA methylation included 6-methyladenine (m6A), 5-methylcytosine (m5C), 1-methyladenine (m1A), 5-hydroxymethylcytosine (h5mC) and pseudouridine (Ψ) 29. Among them, m6A is the most abundant type of methylation modification in eukaryotic mRNA, which regulates gene expression post-transcriptionally 2, 3. Methylation occurs in the *N*6 of adenine within highly conserved RRACH (R: purine; A: m6A; H: non-guanine) region, that is a dynamic modification regulated by methyltransferases (writers), demethylases (erasers), and m6A-binding proteins (readers) (**Figure 1**). Generally, the m6A modification frequently occurs near the translation stop codon and in 3'-UTR of mRNA 30. “Writers” transfer the methyl groups from the donor S-adenosylmethionine (SAM) to the *N*6 position of adenosine 31. Current studies shows that m6A is marked by writer complex, which consists of the METTL3-METTL14 heterodimer and many vital adaptor proteins, WTAP 32, KIAA1429 (also known as VIRMA) 33, RBM15, or its paralogue RBM15B 34, CBLL1 and ZC3H13 35. METTL3 and METTL14 form a complex that facilitates the transfer of methyl groups to the sixth adenine of mRNA. WTAP plays a regulatory role in the recruitment of METTL3-METTL14 heterodimeric enzyme complex to nuclear speckles (NSs) which are involved in RNA transcription, AS and export, although it has no catalytic activity 32. In addition, KIAA1429, RBM15 and ZC3H13 are also part of the MTC and collaborate synergistically even though their different roles in mRNA or ncRNA methylation processes 34-36. METTL16 has been identified as an independent U6 snRNA m6A methyltransferase, distinct from the METTL3-METTL14 complex. Generally, SAM serves as a methyl group donor for cellular methylation reactions. Importantly, METTL16 regulates the SAM cycle through controlling the intron retention of SAM synthetase MAT2A, which necessitates methylation substrate on hairpin1 (hp1) in the 3'-UTR of MAT2A 37. Moreover, METTL16 in the nucleolus regulates rRNA maturation, ribosome biogenesis and mRNA translation, possibly through engaging in rRNA methylation 38. In addition, METTL5 and ZCCHC4 have also been identified as the enzyme that methylates rRNA, 18S and 28S, respectively 39, 40. FTO, ALKBH3 and ALKBH5 are demethylases that catalyze the reversal of m6A modification on different RNA molecules 6, 7, 41. FTO mediates the demethylation of RNA in both the nucleus and cytoplasm, exhibiting cell type-dependent variations and exerting distinct effects on various RNA substrates, including mRNA, snRNA and tRNA 42. ALKBH3 mediates the demethylation of m6A in mammalian tRNA, thereby augmenting protein synthesis 41. ALKBH5 catalyzes m6A elimination on nuclear RNA (mostly mRNA), which is confirmed to colocalize with NSs to regulate nuclear RNA export and alternative splicing 6.

Different types of RNA undergo m6A modification through the regulation of methyltransferase and demethylase despite no sequence changes, thereby dynamically controlling various biological processes. Notably, m6A readers, a class of RNA-binding proteins, recognize and selectively bind to the sites modified by m6A, guiding the fate of target RNA. Currently, known m6A readers mainly include three families, YTH domain-containing proteins (YTHDF1-3, YTHDC1-2), hnRNP protein family (hnRNPA2B1, hnRNPC and hnRNPG) and insulin-like growth factor 2-mRNA binding proteins (IGF2BP1-3) 9, 10, 43. YTHDF1-3 and YTHDC1-2 are canonical m6A readers that share the highly conserved YTH domain, enabling recognition of m6A-modified sites through formation of an aromatic cage; however, they exhibit distinct subcellular location and perform diverse molecular biological functions 44. YTHDF1-3 and YTHDC2 are mainly located in the cytoplasm. YTHDF1 facilitates cap-dependent RNA translation through recruiting eukaryotic initiation translation factor (EIF3) and coupling m6A-modified mRNA to ribosomes 45. Most academic perspectives hold that YTHDF2 accelerates the degradation of m6A-modified mRNA through different pathways. For instance, YTHDF2 can recruit the CCR4-NOT deadenylase complex or HRSP12-RNase P-MRP complex to mediate deadenylation of m6A-modified mRNAs and facilitate their endoribonucleolytic cleavage, respectively 46. Interestingly, YTHDF3 exerts an impact on the translation and degradation of m6A-modified RNAs by cooperating with YTHDF1 or YTHDF2, thereby indicating the synergistic functional interplay among these three YTHDF proteins inside cells 47. Nuclear YTHDC1 actively participates in the intricate processes of RNA splicing and transport through recognizing m6A-modified sites and interacting with splicing factor SRSF3 24, 48. Increasing studies indicate that cytoplasmic YTHDC2 has an impact on RNA degradation and translation. Unlike other readers, YTHDC2 has been demonstrated to perform RNA helicase activity through binding U-rich motifs in 3'-UTR of mRNAs during the mitotic-meiotic transition of mouse germ cells, independent of its m6A-reading capacity 49. HnRNP protein families have a wide function in regulating RNA metabolism and have also been identified as “m6A readers”. It is noteworthy that although some of them do not directly bind to m6A-modified sites, they instead interact with RNA regions proximal to m6A modification via a so-called “m6A-switch” mechanism 50, 51. Through this non-canonical mechanism, m6A induces RNA unfolding and enhances the accessibility of hnRNPC, hnRNPG and hnRNPA2B1 to specific RNA binding sites, thereby contributing to the refinement of their alternative splicing activity 50-52. Besides, hnRNPA2B1 has been implicated in the processing of primary miRNA (pri-miRNA) through its interaction with m6A-modified pri-miRNA and the microRNA Microprocessor complex protein DGCR8 23. Additionally, IGF2BPs are identified as a class of non-canonical m6A readers that augment mRNA stability and translation by specifically binding to m6A-modified sites through their KH domains although the binding affinity of IGF2BPs for m6A-modified RNAs is significantly inferior compared to that exhibited by YTH domains 9, 53. Besides, fragile X mental-retardation protein (FMR1) 54, EIF3 55 and proline rich coiled-coil 2 A (PRRC2A) 56 have also been identified as m6A readers that actively participate in RNA metabolism through distinct molecular mechanism. Collectively, different readers are distributed in distinct subcellular locations for binding to m6A-modified RNAs, thereby orchestrating the process of RNA metabolism 57. Overall, m6A modification plays a pivotal role in regulating the whole processes of RNA metabolism, encompassing transcription, splicing, transport, translation and stability **(Figure 1)**.

Noteworthily, m6A modification and its regulators play a crucial role in a variety of physiological processes, including gametogenesis 58, embryogenesis 59, immune response 60 and bone generation 61. However, maladjustment of m6A modification leads to the emergence and development of diseases 62, such as embryonic lethality 63, infertility 64, immune system dysfunction 13 and tumorigenesis. Intriguingly, emerging evidence suggests that m6A-modified RNAs are enriched within the MLOs or BioMCs, in which they recruit and juxtapose m6A readers, facilitating them to undergo LLPS and to form RNA-protein droplets 19. These m6A-associated MLOs or BioMCs have been demonstrated to effectively regulate RNA metabolism in a coordinated manner (**Figure 1**).

## Overview of RNA-associated membraneless organelles

To maintain various biochemical reactions in order, there are subcellular compartments in eukaryotic cells that isolate biomolecules, including canonical membranous organelles, such as mitochondria, Golgi bodies, endoplasmic reticulum and nuclei. Interestingly, some dynamic organelles or apparatus without surrounding membranes, about 0.2~2 μm in size, are also found in the cells, known as MLOs or BioMCs, which exhibit different morphologies and are enriched with abundant proteins and nucleic acids 20. These MLOs have a liquid-like feature and bear liquid droplets, which are assembled or disassembled depending on the cellular condition and partially external stimuli. Non-covalent interactions between various proteins and nucleic acids serve as driving forces for LLPS, facilitating the formation of MLOs 65. It is worth noting that MLOs play essential roles in diverse cellular processes (**Table 1**), such as chromosome maintenance 66, RNA metabolism 20, protein turnover 67, immune signal transduction 68 and cytoskeleton remodeling 69.

**Figure 2:**
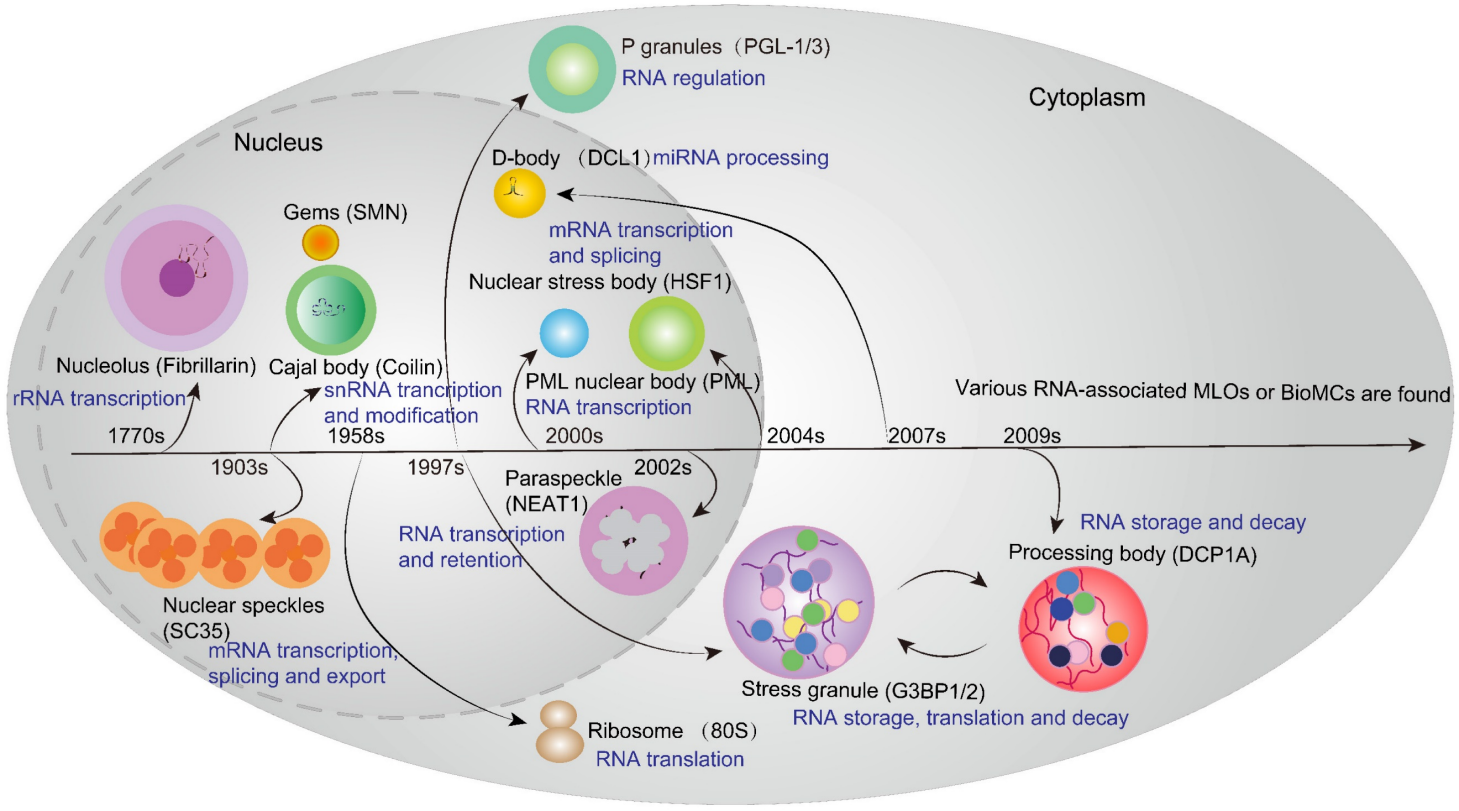
** History of RNA-associated MLOs in Eukaryotes.** Since the discovery of the nucleolus (the first identified MLO) in the 1770s, other RNA-associated MLOs have gradually been identified in eukaryotes, such as Cajal bodies (CBs), nuclear speckles (NSs), paraspeckles, D-body, promyelocytic leukemia protein nuclear bodies (PML-NBs) and nuclear stress body in the nucleus, as well as translational machinery ribosomes, P granules, stress granules (SGs) and processing bodies (PBs) in the cytoplasm. Different MLOs can be marked by key component proteins, such as coilin for CB, SC35 for NSs, and NEAT1 for paraspeckles. These MLOs play a pivotal role in various RNA metabolism processes encompassing transcription, modification, splicing, storage, decay and translation.

With the discovery of the nucleolus (the first identified MLO) in the 1770s, other RNA-associated MLOs, such as Cajal bodies (CBs), Gems, NSs, paraspeckles, promyelocytic leukemia protein nuclear bodies (PML-NBs) and nuclear stress body in the nucleus, as well as stress granules (SGs), processing bodies (PBs) and P granule (*Caenorhabditis elegans*) in the cytoplasm, have gradually been identified in eukaryotes 65 **(Figure 2)**. Their different functions in gene regulation, especially in the RNA metabolism process, are profoundly investigated. For example, the nucleolus and CBs are involved in the biogenesis of rRNA and snRNA respectively, which contributes to the production of macromolecular machineries including ribosomes and spliceosomes. Intriguingly, these two MLOs show the close physical and functional relationship in the nucleus. CBs are the center for the maturation of small nuclear ribonucleoproteins (snRNPs), which are essential for spliceosome assembly, snRNA transcription and modification. Moreover, accumulating studies reveal that CBs are also required for the assembly of small nucleolar ribonucleoproteins (snoRNPs) that are vital for rRNA processing within the nucleolus 70. Notably, PBs in the cytoplasm share some common components with CBs, and communicate and cooperate with each other in the regulation of RNA metabolism. PBs are cytoplasmic MLOs that play an important role in microRNA-induced silencing complex (miRISC) 71, mRNA decay and translation repression 72. The indirect crosstalk between these two independent MLOs can be well illustrated by an example that alternative mRNA splicing and mRNA decay in normal organisms require the balance of LSM1-7 and LSM2-8 complexes in PBs and CBs, respectively 73. It is evident that they share some LSM proteins to play a collaborative role in RNA processing and metabolism. In addition, PBs and SGs are two classical cytosolic MLOs that share some RNPs and mRNAs, and they play a crucial role in translation control and RNA storage. However, SGs only become visible under stressed conditions while PBs are constitutively observed by microscopy. Upon stress, cytosolic translation halts and mRNAs that have been bound to ribosomes or other specific proteins converge to form SGs. Meanwhile, translation-stopped mRNAs that lack binding to translation initiation factors enter the PBs 74. Thus, MLOs may share some components and are assembled when cellular biochemical reaction or condition changes are going to happen. Additionally, it is noteworthy that certain nuclear condensates, such as the nuclear YAP droplet or BRD4 droplet, promote robust downstream gene transcription associated with cellular proliferation and differentiation 75, 76. In addition to the classical RNA-associated MLOs, essential proteins such as cyclic GMP-AMP synthase (cGAS) and P62 can also drive the formation of MLOs, which function as sensors for cytoplasmic DNAs and ubiquitinated proteins, thereby participating in cellular immune response and autophagy 67, 68. However, the aberrant formation or disaggregation of MLOs disrupts biochemical reaction processes and gene regulation, which will lead to the emergence of human diseases, such as Reye's syndrome 66 and Parkinson's disease 77. Moreover, cancer cells employ some MLOs (e.g. SGs) to gain the adaptability in the harsh tumor microenvironment, thereby compromising the efficacy and efficiency of anti-cancer therapies 78. Therefore, restoring or targeting these MLOs may emerge as a promising therapeutic strategy for human diseases.

Extensive studies have demonstrated that multiple folded domains, short linear motifs (SLiMs), IDRs and post-translational modification (PTM) of certain proteins lead to the vulnerability of LLPS to form MLOs 79-81. Notably, post-transcriptional modification, particularly m6A modification, has a great impact on the formation of MLOs, which in turn affects RNA metabolism under both physiological and pathological conditions. In this review, we provide a comprehensive summary of recent studies on the regulation of RNA metabolism processes by m6A-associtated MLOs, with a particular emphasis on the role of m6A modification in driving LLPS in human diseases. Furthermore, we propose that m6A-associated MLOs could serve as predictive markers for disease progression and potential drug targets in the future.

## M6A-associated MLOs participate in RNA metabolism processes

Approximately 25% of mRNA molecules encompass at least one m6A base 82. Accumulating studies demonstrate that this post-transcriptional modification of mRNA exerts regulatory control over its fate, particularly during the processes of RNA transcription, splicing, export, translation and decay. How m6A modification guides these processes is poorly understood. Recently, m6A readers, including YTHDF1-3, have been identified as LLPS proteins to undergo phase separation in cells. Both the N-terminal IDR domain and the C-terminal YTH domain responsible for m6A binding are critical for BioMCs formation 17. Under stress conditions, polymethylated mRNA, not single m6A modified mRNA, enhances phase separation by serving as a multivalent scaffold for binding to YTHDF proteins and juxtaposing their low-complexity domains 82.

Moreover, m6A modification in the nucleus is predominantly mediated by methyltransferase complex (MTC), whose assembly has been reported to be dependent on METTL3 LLPS 83. These condensates partition into distinct endogenous MLOs, such as PBs, SGs and NSs, thereby governing the regulation of RNA metabolism 17, 82, 83. In the following, we will provide a summary of recently found MLOs regulated by m6A modification in RNA metabolism processes** (Figure 1)**.

### RNA transcription

M6A is an important modification involved in regulating chromatin states and transcription dynamics. For instance, METTL3 labels m6A modifications to chromosome-associated regulatory RNAs (carRNAs), such as those overlapping enhancers or super-enhancers. The nuclear reader YTHDC1 specifically recognizes these m6A-marked carRNAs (mainly LINE1 repeats), and subsequently elicits their degradation through the nuclear exosome mediated pathway. Knockout of METTL3 or YTHDC1 in mouse embryonic stem cells enhances chromatin accessibility and activates transcription 84. This study suggests that m6A modification on carRNAs exert an influence on the adjacent chromatin state and downstream transcription. Inconsistently, Lee *et al*. found that the m6A-labeled enhancer RNAs (eRNAs) are pervasively present in human cells. These m6A-modified eRNAs absorb the nuclear m6A reader YTHDC1 to strong enhancers, further stimulating gene activation and transcription 21. Knockdown of YTHDC1 inhibits enhancer and gene transcription instead of altering eRNA stability. Mechanistically, m6A-eRNAs/YTHDC1 interaction facilitates LLPS and promotes the formation of transcriptional activator BRD4 condensates (**Figure 1**). Further experiments confirmed that arginine residues enriched in IDR2 at the C-terminal of YTHDC1 are vital for the formation of its condensate, which further enhanced the formation of BRD4 coactivator condensates 21. This study reveals the critical role of m6A-marked eRNA condensates in crosstalk between epitranscriptome and epigenome, which are reminiscent of nuclear speckles in the nucleus 22. However, they did not rule out whether YTHDC1 plays a role in activating gene transcription through direct binding to promoters. Besides, these studies did not establish a causal relationship between m6A levels, number of m6A sites, identity of eRNAs with condensation formation. Furthermore, their investigation did not provide a comprehensive definition of the condensates or unveil the physiological and pathological role of these m6A-associated super enhancer condensates.

### Pre-mRNA splicing and miRNA processing

AS is an important process mediated by spliceosome that produces different RNA isoforms from the same pre-mRNA, which leads to the diversity of proteins involved in biological processes 85. Progressive evidence suggests that m6A modification and its regulators play a pivotal role in the assembly of splicing condensates, as well as in the biogenesis and recruitment of splicing factors to precisely orchestrate AS 22, 86. The study conducted by Dominissini *et al*. first revealed that m6A affects RNA splicing, as evidenced by differential spliced exons and introns induced by METTL3 knockdown in HepG2 cells, of which most were methylated 87. Notably, acute deletion of METTL3 coupled with nascent RNA profiling only reveal several dozens of AS events, such as alternative 5′ splice sites (A5SS) and intron retention (RI), instead of widespread splicing defects 88, in line with the findings from recent studies showing that m6A deposition onto RNA is mostly occluded by exon-junction complex 88, 89. Thus, nuclear splicing inversely governs m6A methylation at the proximal splice site of pre-mRNA. These findings suggest a reciprocal and synergistic relationship between m6A modification and RNA splicing. NSs within the nucleoplasm are vital MLOs that provide the reaction sites for RNA transcription, splicing and modification 22. Notably, it has been acknowledged that NSs contain MTC core proteins, including METTL3, METTL14 and WTAP, in which splicing factor WTAP is required for recruiting METTL3-METTL14 to NSs 22. Interestingly, METTL3 has been reported to endogenously colocalize with NSs and exhibit self-interaction capabilities, thereby promoting the formation of condensates in nucleus. The presence of nascent RNAs plays a crucial role in facilitating the opto-condensate formation of METTL3. However, mutation of SAM binding sites or treatment with an inhibitor targeting MAT2A (SAM synthetase) abolished or prevented the formation of METTL3 condensates. This study suggests that the dynamic assembly of MTC is largely dependent on METTL3 phase separation, and WTAP may regulate the size of endogenous METTL3 droplet 83. In short, m6A modification is essential for NSs formation.

In addition, YTHDC1, a key m6A reader implicated in AS, was also identified as a pivotal component of NSs. In contrast to the NSs-promoting function of METTL3, YTHDC1 forms YT-Bodies at transcriptionally active sites and adjacent to NSs, thereby delineating the localization of splicing factor SRSF3 within NSs and enhancing its RNA-binding affinity. However, it competitively suppresses these abilities of SRSF10, thereby promoting exon inclusion and repressing exon skipping 48, 90. Moreover, YTHDC1 interacts with three mRNA 3'-end processing factors, namely CPSF6, SRSF3, and SRSF6, to modulate alternative polyadenylation and splicing during mouse oocyte development 91. It seems that YTHDC1 potentially regulates the recruitment of splicing factors to NSs, thereby manipulating AS. Indeed, m6A-eRNA, m6A binding domain and IDR are essential for the formation of YTHDC1 nuclear condensates 21. Consequently, it is plausible that YTHDC1 serves as a scaffold protein for NSs formation and further studies are required to confirm this hypothesis.

Moreover, ALKBH5 and FTO, two m6A eraser proteins, are also found to colocalize with NSs 6, 7. Deficiency of ALKBH5 affects assembly of splicing factor ASF/SF2 and SPRK1 in NSs, causing reduced phosphorylation of ASF/SF2 and disturbed pre-mRNA splicing 6, 11. Interestingly, disturbance of ALKBH5 led to the disappearance of NSs. It is speculated that ALKBH5 may also be a scaffold protein for NS formation since the critical study has confirmed that ALKBH5 LLPS facilitates the formation of BioMCs. The demethylation activity of FTO in the nucleus has a significant impact on pre-mRNAs processing, including exon inclusion and 3'-end processing 92. However, knockdown of FTO did not impact the number of NSs, which suggests that FTO may not be essential for NSs formation but rather serves as an adaptor protein for pre-mRNA splicing through demethylating m6A-modified transcripts and cooperating with other splicing factors in NSs. Furthermore, an unprecedented study conducted by Mauer *et al*. revealed that FTO selectively demethylates the N6,2′-O-dimethyladenosine modification (m6Am) on snRNAs before it happens cap hypermethylation (m7G), thereby exerting further influence on AS 93. SnRNAs are the core spliceosome components involved in pre-mRNA splicing, necessitating a series of modifications to protect them from degradation before being incorporated into small nuclear ribonucleoproteins (snRNP) 94. Thus, FTO may also indirectly affect AS through regulating the assembly of spliceosome.

These above-mentioned studies suggest that m6A core proteins not only regulate m6A methylation or demethylation, but also promote the formation of NSs, the assembly of splicing machinery and participate in NSs-mediated pre-mRNA splicing. NSs share some proteins with other MLOs, such as CBs, which is the assembly compartments of spliceosomal snRNPs before their relocation to NSs. Will m6A modification or regulators also impact the assembly of snRNPs in CBs?

Recent studies have also indicated that m6A is enriched in primary microRNA (miRNA) transcripts, which play a pivotal role in controlling miRNA biogenesis within the cellular nucleus 95. Alarcon *et al*. found that hnRNPA2B1 is a nuclear “reader” of the m6A mark, mediating miRNA processing and AS. Knockdown of hnRNPA2B1 resulted in similar genome-wide AS events observed with METTL3 deficiency. Notably, depletion of hnRNPA2B1 led to the nuclear accumulation of specific pri-miRNAs, also resembling the effects seen with METTL3 knockdown. Mechanistic studies revealed that hnRNPA2B1 can recruit DGCR8, a component of microprocessor complex, to a subset of precursor miRNAs, thereby promoting pri-miRNA processing 23. Heuristically, a recent study found that phase separation of SERRATE promotes the assembly of dicing bodies (D-bodies), which are the miRNA processing MLOs in *Arabidopsis* 96. The low complexity domains (LCDs) present in multiple RNA-binding proteins (RBPs) promote LLPS. Indeed, hnRNPA2B1 has two RRM regions at the N-terminus and a conserved LCD at the C-terminus. Upon cellular stress conditions, hnRNPA2B1 is recruited to SGs 97. It is fascinating to assume that hnRNPA2B1 could serve as the key driver protein for LLPS, facilitating recognition of m6A-modified pri-miRNA and interaction with the microprocessor complex, thereby portioning into condensates involved in pri-miRNA processing, such as D-bodies.

### RNA export

Accumulating evidence suggests that RNA modification, particularly m6A, is intimately associated with RNA export or nuclear retention 98. It has been reported that hyperphosphorylated ASF/SF2, a splicing factor highly enriched in multisite phosphorylation of its C-terminal RS domain, regulates pre-mRNA splicing in NSs while hypophosphorylated ASF/SF2 facilitates the association between the TAP-p15 complex and mRNA cargo to enhance mRNA export and further modulates translation initiation of bound mRNA 99-101. As above mentioned, ALKBH5 regulates pre-mRNA splicing in NSs by promoting assembly of splicing factor ASF/SF2. Interestingly, knockdown of ALKBH5 diminished phosphorylation of ASF/SF2 and promoted the production of methylated transcripts, which facilitates mRNA export to the cytoplasm 6. This suggests that m6A promotes RNA export and ALKBH5-mediated demethylation activity enables selective entry of pre-mRNA into NSs for processing. Upon viral infection, DDX46 recruited ALKBH5 to demethylate m6A-modified host antiviral transcripts, which consequently enhances their nuclear retention, represses cytoplasmic translation and limits interferon production 102. Importantly, ALKBH5 has been reported to promote the assembly of paraspeckles (PSs) through LLPS and demethylating NEAT1, a scaffolding lncRNA for PSs formation, and enhancing its stability 103, 104. Therefore, ALKBH5 may promote the nuclear retention of transcripts through demethylating m6A-modified transcripts and promoting the assembly of nuclear m6A-associated MLOs. On the contrary, YTHDC1 facilitates nuclear RNA export through recognizing or binding to m6A-modified mRNA 24, 105, 106. Mechanistically, the interaction between YTHDC1 and SRSF3 contributes to RNA crowding to nuclear RNA export factor 1 (NXF1), which is essential for efficient export of methylated mRNAs from the nucleus 24. This finding suggests that YTHDC1 is important for substrate RNA selection in the mRNA export pathway. In this nuclear-to-cytoplasmic process, SRSF3 functions as an adapter protein facilitating the export of methylated nuclear mRNAs bound by both YTHDC1 and NXF1, rather than acting solely as a splicing factor 24, 107. Whether this process is carried out in m6A-associated MLOs is unknown. Given the numerous reports highlighting YTHDC1's involvement in nuclear MLOs formation, particularly in RNA transcription and splicing via LLPS, it is intriguing to investigate its potential contribution towards the assembly of RNA transport condensates.

### RNA degradation

Recent studies have demonstrated that m6A modification plays a crucial role in governing mRNA degradation 108, 109. For decades, the complex of YTHDF proteins and m6A-modified mRNAs have been reported to partition into intercellular condensates through LLPS, leading to the formation of cytoplasmic MLOs such as PBs and SGs. PBs are enriched with deadenylase, decapping complex and 5'-3' exoribonuclease, indicating their crucial role in post-transcriptional regulation 72. Evidence indicates that PBs and SGs present “docked” against each other during the assembly of SGs, despite being considered distinct MLOs. This intriguing presentation is a result of the sharing RBPs and mRNAs shuttling between PBs and SGs 74. In particular, YTHDF2 presents the versatile ability to partition into different subcellular compartments, such as PBs under normal conditions and SGs during cellular stress 82. Functionally, YTHDF2 serves as the prototypical m6A reader that actively engages in two canonical mRNA decay pathways. YTHDF2 contains a C-terminal RNA-binding domain known as the YTH domain, for binding to m6A-modified mRNA, and a P/Q/N-rich N terminus (N-YTHDF2), responsible for directing the localization of YTHDF2-mRNA complex to cellular RNA decay sites such as PBs 110. In detail, YTHDF2 promotes the decay of its mRNA targets through recruiting CCR4-NOT deadenylase complex 111. Interestingly, HRSP12, a 14.5 kDa translational inhibitor protein, has been recognized as an adaptor to connect YTHDF2 with RNase P/MRP (endoribonucleases), which subsequently elicits the rapid degradation of YTHDF2-bound RNAs. If m6A-modified RNAs possess HRSP12 binding sites upstream and downstream YTHDF2-binding sites, along with RNase P/MRP-directed cleavage sites, these RNAs will undergo preferential degradation through the RNase P/MRP-mediated endoribonucleolytic pathway coupled to the CCR4-NOT complex-mediated deadenylation pathway. Despite lacking HRSP12 binding sites, YTHDF2 may still interact with HRSP12 and trigger both decay pathways less efficiently 46. Nevertheless, deadenylation is known to precede PB formation, and subsequent 3'-5' exoribonuclease cleavage in exosome is also carried out outside PBs. Thus, the remaining RNA intermediates may be processed by decapping reaction and 5'-3' degradation in PBs. Besides involvement in RNA decay, PBs are also identified as a cytoplasmic hub for untranslated RNA storage 72, 112. Recently, YTHDFs were found to synergistically destabilize mRNAs, irrespective of their m6A modification. Evidently, the triple knockdown of all three members of YTHDF proteins leads to an increased number of PBs and enhanced mRNA stability, independent of methylation status 113. Consequently, these proteins also serve a non-redundant role as scaffolds in maintaining RNA granule formation.

Early embryonic development depends on maternal RNAs and proteins since the zygotic genomes are transcriptionally silent 114. Fragile X mental-retardation protein (FMR1), a ribosome-associated RBP, is present in several cytoplasmic RNP granules, such as PBs, stress granules and neuronal granules, which was found to participate in embryogenesis 54. In *Drosophila*, FMR1 exhibits a preferential binding affinity towards m6A-modified mRNAs, which induces FMR1 granule condensation through recruiting numerous unmodified mRNAs. Specifically, the LC domain initiates the self-assembly of FMR1 BioMCs while KH domains enhance affinity for capturing m6A-modified mRNAs. Importantly, these dynamic m6A-associated FMR1 BioMCs facilitate degradation of maternal RNA and contribute to normal development 54.

Collectively, MLOs or BioMCs formed by YTHDFs, FMR1 and m6A-modified transcripts may provide sites for efficient degradation of redundant RNAs or storage of crucial RNAs under physiological conditions. However, cancer cells can benefit from these “RNA shredders” through promoting the decay of tumor suppressor genes or storing oncogenic transcripts.

### RNA translation

Accumulating studies suggest that m6A modification on RNA translation is multifaceted and complicated, such as recruiting translation initiation factors and interacting with them, promoting ribosome biogenesis, SGs and PBs formation 38, 115, 116. For example, METTL16 colocalizes with the nucleolus, a phase-separated nuclear condensate, and regulates rRNA maturation likely through methylating pre-rRNA prior to processing, which is required for ribosome biogenesis (**Figure 1**). Moreover, METTL16 interacted with specific nucleolar proteins, including NOLC1, TCOF, UBF1/2 and fibrillarin. Knockout of METTL16 resulted in a reduction in the number of nucleoli, indicating its essential role in nucleolus formation 38, 117. However, it remains unconfirmed whether METTL16 can drive nucleoli formation through LLPS due to its possession of multiple RNA-binding sites and a long IDR.

As abovementioned, YTHDF2 primarily participates in RNA decay mediated by PBs. Interestingly, YTHDF1 and YTHDF3 may selectively promote translation through binding to m6A motif. SGs are a group of RNP granules formed under various stress conditions, such as chemicals, UV light, heat or oxygen radical stimulation, which function in controlling RNA translation and degradation 20. Recent studies have revealed that m6A-modified RNAs are also enriched in SGs, and m6A-binding YTHDFs enhance the formation of SGs 17. All three YTHDF proteins are found to colocalize with G3BP1, a classical molecular marker of SGs. However, due to significant differences in their protein sequence within LCDs, YTHDF1/YTHDF3 and YTHDF2 form distinct RNA granules, as evidenced by the co-localization of YTHDF2 with both SGs and PBs. Knockdown of YTHDF1/3, but not YTHDF2, resulted in a substantial reduction in the number of SGs, along with significantly diminished m6A and polyA signals within these granules 17. This finding suggests that YTHDF1 and YTHDF3 may exert selective control over RNA translation in comparison to YTHDF2. Consistently, growing studies show that YTHDF1 and YTHDF3 facilitate translation of many m6A-modified mRNAs, such as EIF3C, ATG2A, ATG14 and FZD7 during tumorigenesis 118-120. In the cytosol, mRNAs undergo dynamic categorization into translation and non-translation pools. A recent study conducted by Shan *et al*. has revealed that m6A modification is an important element switching mRNAs from polysome to PBs, which was mediated by IGF2BP3, a crucial m6A reader in the cytoplasm 25. They also confirmed that decreased m6A modification has no impact on the number of PBs, but it affects mRNA trafficking into PBs. IGF2BP3 directly interacts with core proteins DDX6 and 4E-T in PBs, as well as with TIAR, a core protein in SGs 25. Therefore, IGF2BP3 may facilitate the assembly of m6A-assocaited MLOs involved in mRNA storage and stability during cellular stress. When stress is “unlocked”, some mRNAs bound to initiation factors can be transported into ribosomes for translation. In conclusion, m6A-associated SGs can be a vital translation adaptor under cell stress conditions.

## The role of m6A-associated MLOs in human diseases

It is widely known that maladjustment of RNA metabolism due to aberrant post-transcriptional modification will lead to the emergence of multiple human diseases, including respiratory, neurological, reproductive diseases and tumorigenesis. As abovementioned, under stable or stressful conditions, m6A-modified mRNA is required for the assembly of biomolecular condensates involved in various RNA metabolism processes. Noteworthily, certain MLOs or BioMCs that are induced by aberrant m6A levels or regulators (writers, readers and erasers) have been progressively recognized as pivotal factors driving diverse diseases (**Table 2**).

### Tumorigenesis

**Figure 3:**
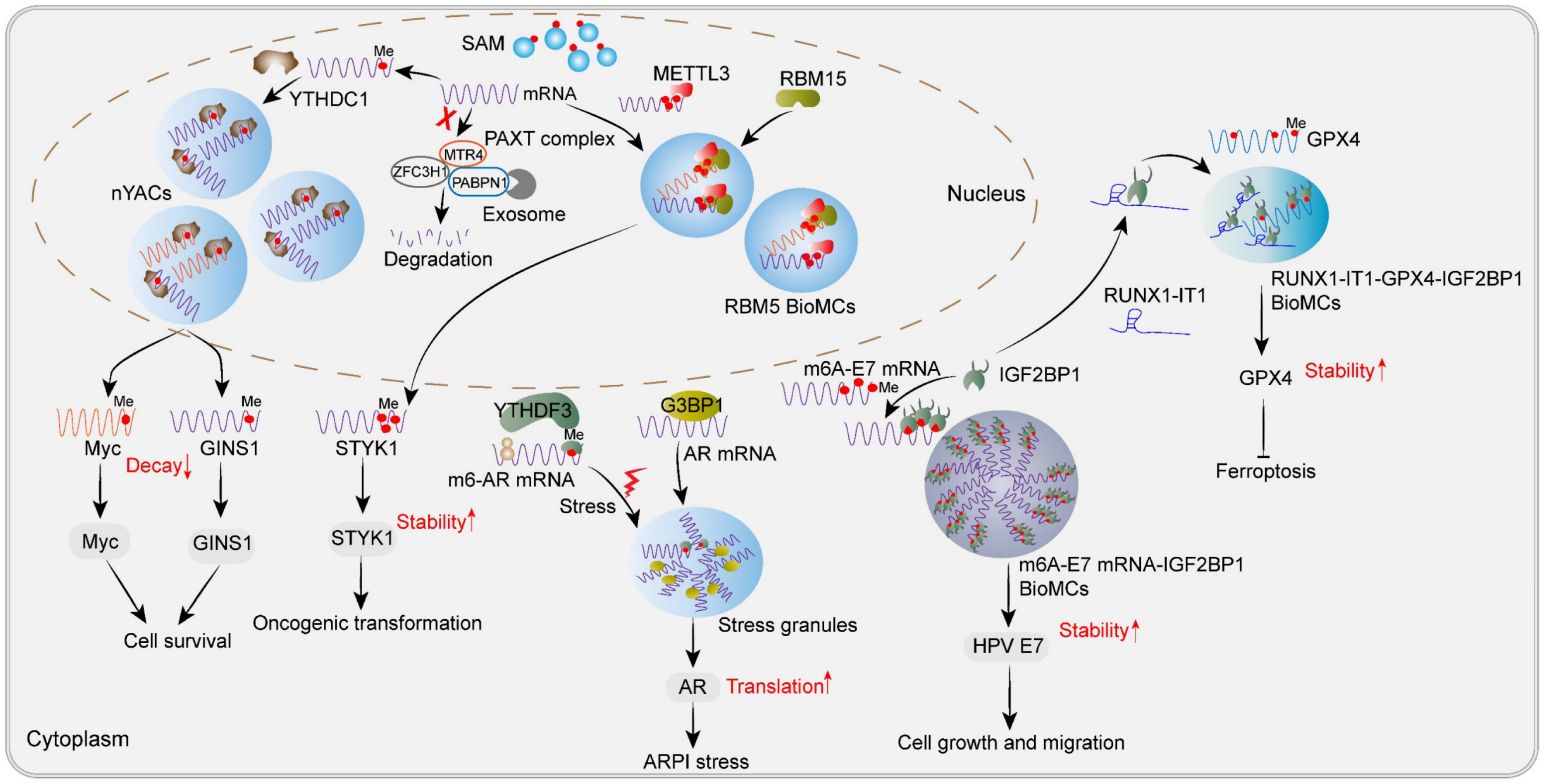
** Newly found m6A-associated MLOs or BioMCs during tumorigenesis.** Nuclear m6A-assocaited MLOs, including nYACs and RBM15 condensates, as well as cytoplasmic IGF2BP1 condensates and SGs, play an oncogenic role in human cancers. nYACs protect oncogenic transcripts *c-Myc* and *GINS1* from nuclear PAXT-exosome mediated RNA decay, thus promoting cancer cell survival in AML. RBM15 recruits METTL3 to methylation compartments through LLPS to methylate *STYK1* and enhance the stability of *STYK1*, thereby inducing oncogenic transformation of NIH3T3 cells. Cytoplasmic m6A-HPV E7 mRNA-IGF2BP1 condensates enhance the stability of HPV *E7* mRNA, promoting cell growth and migration in HPV-associated cancers. In addition, lncRNA RUNX-IT1 can directly bind to IGF2BP1 and promote IGF2BP1 condensates formation, leading to increased occupancy on m6A-modified *GPX4* mRNA and enhancing its stability, thereby inhibiting ferroptosis in breast cancer. SGs induced by AR pathway inhibition (ARPI) play a cell-protective role in prostate cancer. Upon ARPI stress, m6A-modified AR mRNA undergoes LLPS with YTHDF3 while unmodified AR mRNA undergoes phase separation with G3BP1.Upon resolution of stress conditions, translation of AR mRNA occurs regulated by YTHDF3.

Recently, Cheng *et al*. found that m6A is critical for YTHDC1 to drive LLPS and promotes the formation of nuclear YTHDC1-m6A condensates (nYACs). Compared to normal hematopoietic stem and progenitor cells, there was a significant upregulation in the number of nYACs observed in acute myeloid leukemia (AML) cells 26. Functional experiments have confirmed that nYACs are essential for AML cell survival and differentiation control. Further experiments confirmed that both IDR domains and m6A-binding regions of YTHDC1 are vital for nYACs to exert their oncogenic function in AML. Interestingly, 40% and 35% of nYACs were identified as NSs and super-enhancer condensates respectively, which consequently govern RNA abundance and indirect RNA splicing. Mechanistically, nYACs repressed the PAXT-mediated nuclear decay of m6A-modified mRNAs, such as Myc and GINS1 26, which coincides with the fact that YTHDC1 recognizes m6A-modified RNA transcripts in the nucleus and promote their stability and transport 24. Thus, these nuclear bodies induced by m6A-modified mRNA and YTHDC1 LLPS are crucial regulators for tumor cell survival and differentiation repression (**Figure 3**). However, the clinical significance of nYACs in cancer patients remains undisclosed.

In addition, RNA-binding motif protein 15 (RBM15) has been identified as a component of the MTC and functions as a mediator for METTL3 recruitment. It also forms substantial nuclear condensates that exhibit dynamic fusion and fission characteristics 121. Ectopic expression of RBM15 promotes the proliferation and oncogenic transformation of NIH3T3 cells both *in vivo* and *in vitro*. Intriguingly, RBM15 condensates exhibit colocalization with abundant m6A signals, partially interacting with or being embedded into the NSs, while showing no association with other nuclear MLOs, including PML bodies, CBs, paraspeckles, nucleoli and chromatin.

Though RRM domain, IDRs in the N-terminal and SPOC in the C-terminal truncates tend to form smaller droplets, concrete domains responsible for RBM15 LLPS have not been dissected yet. Subsequent proteomics, transcriptomics and RNA methylomics experiments indicated that RBM15 condensates may represent a novel class of subnuclear MLOs involved in facilitating m6A deposition on oncogenic transcripts, such as serine/threonine/tyrosine kinase 1 (STYK1), thereby enhancing their stability 121. Other known stability enhancers for m6A-modified transcripts, IGF2BP3 and YTHDC1, were also found in RBM15 condensates 121. Collectively, YTHDC1 or RBM15 condensates dispersed in the nucleus have made great contributions to the stabilization of the oncogenic transcripts, thereby promoting tumorigenesis, and future anti-tumor strategies can inhibit the formation of these condensates.

Excitingly, inhibiting some m6A-associated BioMCs showed good efficacy in cancer cells and mouse allogeneic tumor model 122. Human papillomavirus (HPV)-induced carcinogenesis relies heavily on virus early protein 7 (E7), which makes E7 a promising drug therapy target 123. Recently, Wang *et al*. found that m6A-modified E7 mRNA-IGF2BP1 condensates, which are oncogenic in nature, stabilize E7 mRNA but exhibit thermal instability and form insoluble aggregates. Clearly, m6A modification plays a crucial role in facilitating the stability of E7 mRNA and enhancing the assembly of oncogenic condensates through binding to IGF2BP1, as demonstrated by the negligible impact on heat-induced condensate formation upon deletion of IDR region of IGF2BP1. Upon prolonged heat stress treatment, these condensates undergo a phase transition and form insoluble aggregates, which are subsequently degraded by the ubiquitin-proteasome system. Noteworthily, heat stress selectively destabilizes E7 mRNA and downregulates E7 protein expression without affecting other m6A-modified transcripts such as *CREBBP*, *RAB21*, and *FBXO41*. Significantly, daily heat treatment inhibited the malignancy of HPV16-positive cells both *in vitro* and *in vivo* through lowering E7 mRNA expression along with IGF2BP1 122. Thus, targeting m6A-E7 mRNA-IGF2BP1 condensates could be a promising therapeutic strategy for HPV-associated cancers, highlighting the clinical potential of anti-tumor therapy by focusing on m6A-associated MLOs. Most recently, Wang *et al*. found that RUNX1 intronic transcript 1 (RUNX1-IT1), known as a tumor driver lncRNA in breast cancer, directly interacts with IGF2BP1 and partitions into BioMCs, thereby occupying and stabilizing glutathione peroxidase 4 (GPX4) mRNA, and then promoting GPX4 protein expression while repressing ferroptosis 124. Rapid clustering of IGF2BP1 has been recently identified as an initiator for SGs assembly upon osmotic shock. The KH3/4 di-domain and an IDR of IGF2BP1 have been confirmed to play crucial roles in mediating this clustering process 125. Deletion of the KH3/4 domain led to the disappearance of these IGF2BP1 condensates 124. However, the specific binding region between IGF2BP1 and RUNX1-IT1 remains unclear. Overall, it is proposed that IGF2BP1 acts as a phase-separated protein driving the formation of m6A mRNA-stabilized MLOs during tumorigenesis, and future anti-tumor therapy may target these IGF2BP1 condensates through inhibiting IGF2BP1 LLPS or dissolving them. Further studies can isolate these MLOs for more comprehensive RNA and proteomic analysis to identify additional potential targets.

Androgen receptor (AR) pathway inhibition (ARPI) triggers strong and lasting responses in advanced prostate cancer 126. Recently, Syam *et al*. found that m6A-modified AR mRNA undergoes liquid-liquid phase separation with YTHDF3, while unmodified AR mRNA undergoes phase separation with G3BP1 in response to ARPI-induced stress in prostate cancer (PCA). These processes facilitate the formation of SGs and drive rapid adaptive changes that confer protection to PCA cells against ARPI-induced stress by regulating the translation of AR mRNA. Inhibiting this adaptive response through knockdown of YTHDF3 or G3BP1 makes PCA cells vulnerable to ARPI stress 127. This study suggests that inhibiting m6A modification of AR mRNA or YTHDF3 could serve as potential therapeutic strategies for ARPI-based therapies in PCA. In contrast to the translational regulatory role of YTHDF3, Y-box binding protein 2 (YBX2) forms stable cytoplasmic condensates through recruiting YTHDF2 and promotes the degradation of m6A-modified HSPA6 mRNA in endometrial adenocarcinoma-derived Ishikawa cells. The cold-shock domain (CSD) and mRNA-binding domain of YBX2 are responsible for recruiting both YTHDF2 and mRNA to LLPS 128. These cytoplasmic condensates may protect cancer cells from chemotherapy.

Collectively, m6A-associated MLOs, including nYACs, RBM15 condensates, IGF2BP1 condensates, SGs and YBX2-YTHDF2 condensates, ensure stable oncogenic mRNA metabolism by regulating RNA decay, nuclear export, stability and translation in cancer cells (**Figure 3**). Some therapeutics, such as targeting the m6A regulator, dissolving the condensates or inhibiting m6A modification of specific mRNA, could be explored as promising anti-cancer strategies.

### Reproductive diseases

Recently, an increasing body of literature has emphasized the crucial role of m6A in RNA metabolism of reproductive systems, including embryo implantation 129, spermatogenesis 11 and embryonic development 12. For example, male mice with ALKBH5 knockout exhibited elevated levels of m6A in mRNA, which was associated with reduced fertility attributed to compromised spermatogenesis. Mechanistically, ALKBH5 regulates mRNA export, stability and the assembly of RNA splicing factor in NSs 6. The demethylation activity of ALKBH5 and its interaction with splicing factors are crucial determinants of ALKBH5's role in regulating RNA metabolism in NSs. Demethylation activity of ALKBH5 in the NSs of spermatocytes and round spermatids is also necessary for accurate splicing and the production of longer 3'-UTR mRNAs 11. Overall, we suspect that maintaining physiological level of ALKBH5 is essential for RNA metabolism in germ cells; however, aberrant dysfunction of ALKBH5 may lead to male infertility. Nevertheless, there is currently a lack of clinical evidence supporting this hypothesis and the specific protein regions responsible for positioning ALKBH5 in NSs have not been dissected yet.

The m6A modification and enzymes also play a critical role in normal embryogenesis and maternal RNA degradation. YTHDC1, a well-known protein involved in LLPS, forms nuclear granules to regulate RNA metabolism. Nuclear YTHDC1 has been confirmed to regulate alternative polyadenylation and splicing through interacting with CPSF6, SRSF3 and SRSF7, which is crucial for mouse embryo viability and germline development. However, knockout of YTHDC1 in oocytes leads to the formation of large novel RNA-containing granules that may comprise abnormal processed transcripts 91. Like PBs, these cytoplasmic granules may serve as a repository for misprocessed RNA transcripts. Indifferently, in *Drosophila*, loss of maternal FMR1, a novel m6A reader, not YTHDF or YTHDC, resulted in embryonic lethality. Mechanistically, FMR1 could form dynamic condensates with m6A-modified RNA molecules, which controls maternal mRNA decay and translation in an m6A-dependent manner 128. Thus, these m6A-associated BioMCs play a crucial role in the processes of gametogenesis and embryogenesis, and ALKBH5 and YTHDC1 are indispensable for RNA processing mediated by NSs in mammals.

In summary, m6A-modified mRNA facilitates the formation of MLOs associated with correct RNA metabolism in reproductive organs. However, aberrant expression levels of m6A writer, reader or eraser proteins may affect the formation and function of these m6A-associated MLOs, leading to infertility or embryonic lethality. Thus, modulating m6A levels to restore the integrity of m6A-associted MLOs could represent a promising strategy for addressing infertility or promoting embryo survival. However, the lack of clinical significance makes this hypothesis a long way from being realized.

### Neurological diseases

Aberrant expression of YTHDFs and disturbed m6A modification have been documented to contribute to the pathogenesis of different human neurological disorders 130. Given the pivotal role of YTHDFs in SGs formation, it is meaningful to elucidate whether SGs triggered by a combination of YTHDFs and stress conditions exert an impact on disease processes. It is widely acknowledged that the microtubule-associated protein tau plays a crucial role in maintaining the stability of microtubules in healthy homeostasis. However, tau oligomerization and hyperphosphorylation accumulate within the somato-dendritic arbor upon stress or disease challenging, which accelerates the progression of tauopathy, such as Alzheimer's disease (AD) 131. Tau oligomerization propagates tau pathology and exhibits extensive co-localization with cytoplasmic TIA1-positive SGs, as evidenced by co-localization with TIA1, PABP and eIF3η 132. Increasing evidence indicates that LLPS and protein aggregates play pivotal roles in SGs formation, particularly in neurodegenerative diseases like tauopathy. Furthermore, RBPs such as TDP-43 (TAR-binding protein of 43 kDa), TLS/FUS (Translocated in LipoSarcoma/Fused in Sarcoma) and hnRNPA2B1, are known to translocate from the nucleus to the cytoplasm and localize within SGs 133. Among them, a prion-like domain (LCD) mutation of hnRNPA2B1, an indirect m6A reader, has been reported to enhance aggregation and drive itself to be sequestrated in SGs in multisystem proteinopathy (MSP). Notably, hnRNPA2B1 is merely located in the nucleus in normal muscle but it accumulates at cytoplasmic inclusions in some muscle fibers of MSP patients 134, 135. Thus, hnRNPA2B1 may participate in SG formation through LLPS in human neurological diseases.

Recently, Jiang *et al*. found that light-induced tau oligomerization (o-tau) promotes the assembly and neurotoxicity of SGs, concomitant with the cytoplasmic translocation of hnRNPA2B1 in cultured neurons. Further mechanistic experiments confirmed that tau phosphorylation enhances its interaction with hnRNPA2B1 via accelerating oligomerization. HnRNPA2B1 was proved to be the key bridge protein connecting o-tau and m6A modified transcripts. Evidently, knockdown of hnRNPA2B1 drastically attenuated the formation of SGs, as suggested by reduced PABP and EIF3η, and ameliorated tau-mediated neurodegeneration in both neurons and tau mice, as suggested by decreased levels of o-Tau, m6A and cleaved caspase-3. Thus, the oTau-hnRNPA2B1-m6A complex enhances SGs formation and regulates the translational response to stress conditions. Importantly, hnRNPA2B1 was found to co-localize with both o-tau and cytoplasmic m6A in human AD samples, indicating the clinical significance for m6A-associated SGs 27. However, the specific region within hnRNPA2B1 that drives toxic SG formation remains elusive. The LCD in C-terminal and RNA-binding domain may be responsible for SGs formation in AD. Further investigations are required to validate this hypothesis.

TDP43, an hnRNP, is also a nuclear RNA-binding protein that regulates RNA metabolism. However, cytoplasmic mislocalization and aggregation of TDP43 are frequently presented in neurons of amyotrophic lateral sclerosis (ALS) patients 136, 137. Recently, Michael McMillan and coworkers found a significant enrichment of m6A-modified transcripts in post-mortem samples obtained from ALS patients. Interestingly, methylated RNAs and YTHDF2 foci are found to be colocalized with TDP43 pathology in the spinal cord and frontal cortex of ALS patient, suggesting that aberrant cytoplasmic aggregates of TDP43 may predominantly influence m6A-marked RNA metabolism. Furthermore, knockout of Ythdf2 alleviated TDP43-related neural toxicity in rodents, whereas overexpression of Ythdf2 led to fatal outcomes 138. In TDP43-overxpressing human iPSCs-derived neuron, a wide RNA destabilization was observed. Considering the recognition of m6A-labeled RNA in PBs and its subsequent degradation mediated by YTHDF2, it is worth investigating whether TDP43-binding m6A transcripts recognized by YTHDF2 undergo degradation in PBs. Moreover, whether YTHDF2 enhances LLPS of TDP43-m6A binding complex is related to the instability of neuroprotective transcripts requires further clarification.

In addition, aberrant cytoplasmic aggregation of TLS/FUS, a nuclear RNA-binding protein, is also implicated in the pathogenesis of ALS. Previous studies have emphasized that post-translational modifications, such as arginine methylation and phosphorylation of TLS/FUS's IDR region, influence LLPS and subsequent aggregation 139, 140. Unexpectedly, Ryoma *et al*. proposed that TLS/FUS might serve as a potential m6A reader candidate even though it does not contain YTH domain. They further found that m6A modified short RNA fragments effectively suppress LLPS of TLS/FUS or its mutants. Additionally, treatment with m6A modified short RNA treatment derived from *pnc*RNA-D obviously attenuated cytoplasmic TLS/FUS foci induced by sorbitol, a type of hyperosmotic stress, in cultured neural cells, thereby promoting cell viability. Moreover, there was a slight reduction in SGs, as TLS/FUS also exhibited colocalization with SGs 141. This study is reminiscent of the fact that polymethylated mRNA promotes LLPS rather than a single m6A methylated mRNA 82. Thus, treatment with m6A modified short RNA fragments may be a strategy to inhibit MSP. Further studies are required for investigating the therapeutic efficacy of m6A-modified RNA fragments both *in vitro* and *in vivo*.

### Respiratory diseases

Accumulating evidence indicates that m6A modification is involved in anti-viral or -bacterial immune response 13, 142, 143. Notably, RNA metabolism associated with m6A in some respiratory diseases also depends on the formation of MLOs. In allergic airway inflammation, allergens promote the LLPS of YTHDF1 and trigger the formation of a complex comprising dimeric YTHDF1 and CLOCK mRNA, which localizes within SGs. This stress adaptive process induced by YTHDF1 LLPS enhances the translation of CLOCK mRNA, thereby promoting the generation of NLRP3 inflammasome and secretion of IL-1β, leading to airway inflammatory responses 144. This study suggests that YTHDF1 condensates play a vital role in the pathogenesis of allergic airway inflammation through regulating CLOCK mRNA translation. Moreover, aberrant upregulation of YTHDF1 was observed in asthma patients compared to control subjects 144, therefore targeting YTHDF1 condensates may be a clinical strategy for improving allergic airway inflammation.

*Mycobacterium tuberculosis* (*M. tuberculosis*) is a highly successful intracellular pathogen responsible for causing tuberculosis (TB), resulting in 10 million active cases and 1.5 million deaths annually 145. Upon *M. tuberculosis* infection, METTL14 LLPS promoted the formation of MTC complex and mediated the methylation of NOX2 mRNA in macrophages. However, EsxB, a *M. tuberculosis*-specific secretory protein encoded by an RD-1 region, repressed METTL14 LLPS through inhibiting p38-mediated phosphorylation of Thr72 within the IDR domain of METTL14, thereby disrupting the m6A methylation of NOX2 mRNA and its subsequent interaction with IGF2BP1, leading to NOX2 mRNA instability. Consequently, this results in increased intracellular survival ratio of *M. tuberculosis* by diminishing ROS levels. Importantly, enhanced T72 phosphorylation of METTL14 was clinically associated with Nox2 mRNA expression level and ROS production in primary TB patients 142. Therefore, METTL14 condensates in macrophages may contribute to the eradication of *M. tuberculosis* through catalyzing m6A methylation.

## Concluding remarks and prospect

Over the past few decades, a growing body of evidence has suggested that m6A modification plays an essential role in the physiological and pathological processes of eukaryotes. Importantly, m6A-modified RNA and its regulators such as METTL3, YTHDF1-3 and YTHDC1 were proven to be enhancers for MLOs formation. These m6A-associated MLOs run through the life of mRNA, engaging in RNA transcription, splicing, export, decay and translation; however, maladjustment of which causes reduced fertility, enhanced cancer cell growth, tauopathy and respiratory disorders. In parallel, disorders of RNA metabolism will follow, encompassing aberrant alternative splicing, subcellular mislocalization of RNAs and augmented stability of oncogenic transcripts. Although many studies have primarily demonstrated that m6A modification in different human diseases, it is imperative to acknowledge that m6A-associated MLOs serve as pivotal biochemical reaction sites and exert substantial regulatory roles in disease progression. Thus, m6A-associated MLOs may be clinical diagnostic markers and attractive drug targets despite the lack of authoritative reports establishing a direct correlation between m6A-associtaed MLOs and disease severity. Mutation of LCD in m6A regulators, such as hnRNPA2B1, results in ectopic production of SGs, which leads to multisystem proteinopathy. It is meaningful to investigate the correlation between the mutation of m6A regulators in the LCD and related human diseases, particularly cancer. Alteration of m6A modification through targeting the LLPS driven region in m6A writer, reader or eraser may be an effective strategy for correcting disorders of RNA metabolism induced by m6A-associated MLOs in human diseases.

## Figures and Tables

**Table 1:** The engaged cell process or function of representative MLOs or BioMCs in eukaryotes.

Localization	Name	Core LLPS protein/RNA	Specific cellular environment	Cellular processes/function	Reference
Nucleus	Nucleolus	Fibrillarin	No	Regulates rRNA transcription, processing and modification, thereby facilitating the assembly of ribosome	20, 65
	Nuclear speckle	SRRM2, MALAT1	No	Regulates pre-mRNA synthesis and splicing, mRNP maturation and RNA transport	20, 22
	Paraspeckle	NEAT1, NONO, SFPQ	No	Regulates RNA retention, transcription and stability	20, 104
	Cajal body	Coilin, SMN	No	Regulates pre-snRNA transcription, modification and snRNP assembly	70
	Gem	SMN	No	Promotes maturation of snRNPs	20, 65
	Nuclear stress body	HSATIII lncRNA, HSF1	Thermal stress	Regulates pre-mRNA splicing upon thermal stress	20
	PML nuclear body	PML	No	Regulates chromosome association and transcription	20, 65
	D body	DCL1	No	Promotes miRNA processing	96
	Heterochromatin condensates	HP1, MECP2	No	Regulates chromosome maintenance, segregation and transcriptional silencing	66
	YAP condensates	YAP	Hyperosmotic stress	Activates downstream gene transcription involved in cell proliferation	75
	Super enhancer condensates	BRD4, MED1	No	Drives robust gene transcription involved in cell identity	76
Cytoplasm	Stress granule	G3BP1/2	Different cell stress	Regulates RNA storage, decay and translational control upon various stress condition	74
	Processing body	DCP1A, LSM14A	No	Regulates RNA storage and decay	71, 72
	P62 body	P62	No	Ensures effective autophagic degradation of ubiquitinated proteins and damaged organelle clearance	67
	cGAS condensates	GAS	Cytoplasmic DNA arising	Activates innate immune signaling	68
	D-granules	DIAPH3	Different cell stress	Regulates actin cytoskeletal remodeling upon various cell stress condition	69

**Table 2:** The role and mechanism of m6A-associated MLOs in human diseases. NA represents not available.

M6A regulator	Protein domain driving LLPS	MLOs	Subcellular location	Disease	Function	RNA metabolism processes	Reference
YTHDC1	N-terminal IDRs and C-terminal YTH domain	YTHDC1-m6A condensates	Nucleus	Acute myeloid leukemia	Regulate cell growth and differentiation control	Protect oncogenic transcripts from decay by the PAXT complex	26
RBM15	RRM domain, IDRs at N-and SPOC at C-terminal	RBM15-m6A-condensates	Nucleus	Tumorigenesis	Promote oncogenic transformation of normal cell	Promote the stability of oncogenic transcripts, such as STYK1	121
IGF2BP1	M6A binding domain	HPV E7-IGF2BP1 condensates	Cytoplasm	HPV-associated cancers	Promote cell growth and migration	Stabilize HPV E7 mRNA	122
IGF2BP1	KH3/4 domain	RUNX-IT1 -IGF2BP1-GPX4 condensates	Cytoplasm	Breast cancer	Promote tumorigenesis	Stabilize GPX4 mRNA	124
YTHDF3	M6A binding domain	SGs	Cytoplasm	Prostate cancer	Promote stress adaptive cell survival	Regulate the translation of AR mRNA	127
YBX2	CSD domain and mRNA binding domain	YBX1-YTHDF2 condensates	Cytoplasm	Endometrial cancer	Delay the cell proliferation and may promote chemotherapy resistance	Promote the degradation of HSPA6 mRNA	128
ALKBH5	NA	NSs	Nucleus	Men infertility	Deficiency of ALKBH5 leads to the impaired fertility	Promote accurate RNA splicing and impact RNA export	6, 11
YTHDC1	NA	Large RNA-containing granules	Cytoplasm	Developmental defect	Deficiency of YTHDC1 leads to impaired gametogenesis and embryogenesis	Regulate alternative polyadenylation and splicing	91
HnRNPA2B1	Prion-like domain and m6A-binding region	Stress granules (oTau-hnRNPA2B1-m6A condensate)	Cytoplasm	AD	Promote tau fibrillization, nuclear membrane disruption, and progressive neurodegeneration.	Control RNA translation	27
TDP43/YTHDF2	NA	TDP43-YTHDF2-m6A condensates	Cytoplasm	ALS	Promote TDP43-mediated toxicity	Enhance RNA destabilization	138
TLS/FUS	IDR domain	TLS/FUS condensates	Cytoplasm	ALS	Promote the cell viability	M6A-modified short RNA fragments inhibit the LLPS of TLS/FUS	141
YTHDF1	IDR domain and prion-like domain	SGs	Cytoplasm	Asthma	Promote airway inflammatory responses	Enhance the translation of CLOCK mRNA	144
METTL14	Thr72 phosphorylation at IDR domain	METTL14 condensates	Nucleus	Tuberculosis	Inhibit intracellular survival of *M. tuberculosis*	Enhance m6A modification of Nox2	142

## Abbreviations

AD: Alzheimer's disease
ALS: amyotrophic lateral sclerosis
AML: Acute myeloid leukemia
AR: Androgen receptor
ARPI: Androgen receptor pathway inhibition
AS: Alternative splicing
BioMCs: Biomolecular condensates
carRNAs: Chromosome-associated regulatory RNAs
CBs: Cajal bodies
CSD: Cold-shock domain
D-bodies: Dicing bodies
E7: Virus early protein 7
EIF3: Initiation translation factor 3
eRNAs: enhancer RNAs
FMR1: Fragile X mental-retardation protein 1
GPX4: Glutathione peroxidase 4
hp1: Hairpin1
HPV: Human papillomavirus
IDRs: Internally disordered regions
LCD: Low complexity domain
LLPS: Liquid-liquid phase separation
M. tuberculosis: Mycobacterium tuberculosis
m6A: N6-methyladenosine
miRISC: microRNA-induced silencing complex
MLOs: Membraneless organelles
MSP: Multisystem proteinopathy
MTC: Methyltransferase complex
ncRNAs: Noncoding RNAs
NSs: Nuclear speckles
NXF1: Nuclear RNA export factor 1
nYACs: Nuclear YTHDC1-m6A condensates
O-tau: Tau oligomerization
PAXT: PolyA tail exosome targeting complex
PBs: Processing bodies
PCA: Prostate cancer
PML-NBs: Promyelocytic leukemia protein nuclear bodies
PTM: Post-translational modification
RBM15: RNA-binding motif protein 15
RBPs: RNA-binding proteins
RUNX1-IT1: RUNX1 intronic transcript 1
SAM: S-adenosylmethionine
SGs: Stress granules
SLiMs: Short linear motifs
snRNPs: Small nuclear ribonucleoproteins
TB: Tuberculosis
TDP-43: TAR-binding protein of 43 kDa
TLS/FUS: Translocated in LipoSarcoma/Fused in Sarcoma
YBX2: Y-box binding protein 2
YTH: YT521-B homology
